# COMP Report: CPQR technical quality control guidelines for CyberKnife^®^ Technology

**DOI:** 10.1002/acm2.12263

**Published:** 2018-01-26

**Authors:** Eric Vandervoort, Horacio Patrocinio, Tom Chow, Emilie Soisson, Dominic Béliveau Nadeau

**Affiliations:** ^1^ Department of Medical Physics The Ottawa Hospital Cancer Centre Ottawa ON Canada; ^2^ Department of Medical Physics McGill University Health Centre – Glen Site Montreal QC Canada; ^3^ Department of Medical Physics Juravinski Cancer Centre Hamilton Ontario Canada; ^4^ Département de Physique Médicale Département Radio‐Oncologie Hôpital Notre‐Dame Montréal QC Canada

**Keywords:** CyberKnife Radiosurgery System, quality control guidelines, radiation treatment therapy equipment, robotic radiosurgery, stereotactive ablative radiotherapy

## Abstract

The Canadian Organization of Medical Physicists (COMP), in close partnership with the Canadian Partnership for Quality Radiotherapy (CPQR) has developed a series of Technical Quality Control (TQC) guidelines for radiation treatment equipment. These guidelines outline the performance objectives that equipment should meet in order to ensure an acceptable level of radiation treatment quality. This particular TQC contains detailed performance objectives and safety criteria for CyberKnife^®^ Technology. The quality control recommendations in this document are based upon previously published guidelines and the collective experience of all Canadian sites using this technology. This TQC guideline has been field tested at the newest Canadian CyberKnife installation site and includes recommendations for quality control of the Iris™ and InCise™ MLC collimation systems.

## 1 INTRODUCTION

The Canadian Partnership for Quality Radiotherapy (CPQR) is an alliance among the three key national professional organizations involved in the delivery of radiation treatment in Canada: the Canadian Association of Radiation Oncology (CARO), the Canadian Organization of Medical Physicists (COMP), and the Canadian Association of Medical Radiation Technologists (CAMRT). Financial and strategic backing is provided by the federal government through the Canadian Partnership Against Cancer (CPAC), a national resource for advancing cancer prevention and treatment. The mandate of the CPQR is to support the universal availability of high quality and safe radiotherapy for all Canadians through system performance improvement and the development of consensus‐based guidelines and indicators to aid in radiation treatment program development and evaluation.

This document contains detailed performance objectives and safety criteria for CyberKnife^®^ Technology. Please refer to the overarching document Technical Quality Control Guidelines for Canadian Radiation Treatment Centres^1^ for a programmatic overview of technical quality control, and a description of how the performance objectives and criteria listed in this document should be interpreted. The development of the individual TQC guidelines is spearheaded by expert reviewers and involves broad stakeholder input from the medical physics and radiation oncology community.^2^

All information contained in this document is intended to be used at the discretion of each individual center to help guide quality and safety program improvement. There are no legal standards supporting this document; specific federal or provincial regulations and licence conditions take precedence over the content of this document.

## 2 SYSTEM DESCRIPTION

In recent years, stereotactic ablative radiosurgery (SABR) has moved from using rigid frames fixed to a patient's skull to the use of noninvasive frameless techniques requiring in room image guidance which are capable of treating extracranial targets. One such system is the CyberKnife^®^ from Accuray Inc. (Sunnyvale, CA, USA) which consists of a compact linear accelerator mounted to an industrial robotic arm. The CyberKnife^®^ system delivers highly conformal radiation doses by delivering multiple radiation fields from many different noncoplanar directions. This is allowed for by the flexibility of the robotic arm and small size of the linac.

The central axes of these beams may share a common point of intersection (isocentric). This type of delivery provides highly conformal spherically shaped radiation dose distributions similar to those delivered using arc therapy with cones on a conventional linac. However, the vast bulk of CyberKnife^®^ treatments use many nonisocentric beams with nonintersecting central axes to treat arbitrarily shaped tumors. For complex targets being treated with circular collimators, this can result in plans with 80–200 beams and tens of thousands of total monitor units per plan.

The most recent generation of the CyberKnife^®^ system has three different secondary collimator systems. The first are the fixed collimators, consisting of 12 circular collimators with nominal diameters from 5 to 60 mm projected at 800 mm from the source. The second is the Iris™, a 12‐sided (two banks of six) regular polygonal variable sized collimators, which in its clinical implementation is restricted to the same equivalent field sizes as the fixed collimators. Use of this collimator decreases treatment time by allowing for changing field sizes and beam directions at each position the robot places the MV photon source (refered to as node positions). The final collimation system is the InCise™ multileaf collimator (MLC) consisting of 41 pairs of 2.5 mm wide leaves as projected at 800 mm from the source, each leaf capable of full interdigitation and over‐travel. The maximum field size of this collimator is 120 mm × 102.5 mm.

The CyberKnife^®^ radiosurgery system uses two orthogonal kilovoltage x‐ray generators and two amorphous silicon flat panel digital detectors for image guidance. CyberKnife^®^ employs several different algorithms to identify the target position in the x‐ray data including skull and spine tracking based on x‐ray contrast of bony anatomy; internally implanted fiducial tracking and tracking based on x‐ray contrast differences between solid tumors and surrounding lung tissue. The system can also compensate for respiratory motion in real time using a predictive algorithm for extracranial treatments. A predictive correlation model is created relating the internal motion of the target to external breathing motion. The external breathing motion is based on the positions of external markers (LED‐based, fiber optic tracking markers) located on the patient's exterior as measured using a stereoscopic camera system. The internal motion is based on the positions of fiducials (referred to as Synchrony^®^ motion tracking), or on the position of a lung tumor itself (referred to as Xsight^®^ Lung Tracking) or on the location of vertebral bodies (for respiratory compensated prone spine treatments). The robotic arm dynamically changes the direction of the linac central axis pointing it to the predicted location of the tumor throughout treatment while the beam is on. All treatments and quality control tests employing respiratory compensation should be observed carefully, listening for unusual noises or vibrations which may indicate problems with robot mastering, robot motion braking, or high levels of noise for the optical marker tracking system.

Comprehensive quality assurance guidelines for robotic radiosurgery were published by the American Association of Physicists in Medicine (AAPM)^3^ in 2011. These guidelines provided QA recommendations for all CyberKnife^®^ tracking algorithms presently available but did not address the use of the Iris or InCise collimation systems. Most of the quality control recommendations in that report have been included in this document with minor modifications based on a consensus between Canadian cancer centers which presently use the technology. This document also includes quality control for the Iris™ and InCise™ MLC collimation systems but, like the AAPM task group report, acknowledges that many issues remain that require further research and development. Some of the quality control tests in both documents are part of the vendor recommended preventative maintenance program. In most centers, these tasks are performed by field service engineers from Accuray. Some tests are performed routinely while others only following hardware or software upgrades. These tests and procedures also evolve as the technology changes. The vendor has a responsibility to clearly communicate changes to its users and provide them with a means of accessing data from individual system components as necessary for quality control testing. It is the responsibility of the medical physicist to provide informed support for this work and adequate return to service testing for all service events. A comprehensive but practical routine quality assurance program for all aspects of this system is required to ensure the accurate and safe delivery of radiation for this unique system Tables 1, 2, 3, 4.

**Table 1:** Daily quality control tests

Designator	Test	Performance	
		Tolerance	Action
Daily			
DL1	Emergency robotic arm motion stop circuit (if present)	Functional	
DL2	Robotic arm collision detection interlocks	Functional	
DL3	Visual check of beam laser and a standard floor mark	n/a	1 mm
DL4	Accelerator output	2%	3%
DL5	Automated quality assurance test (alternate daily between fixed and Iris™ collimators and the InCise™ MLC)	0.75 mm in any direction	1 mm radial
DL6	Modified picket fence field tests for defocused MLC	Visual inspection of junctions	

**Notes on daily tests**	
DL1	For robotic arm radiosurgery units, the emergency motion off button at the console should be included in the circuit test. It should also be verified that the beam is interrupted when this button is engaged.
DL2	The collimator assembly collision detector is the only mechanical interlock placed on robot motion and should be verified daily. Nonstandard patient setups and unusual treatment locations should be verified on a case‐by‐case basis by observing the patient plan delivery in demonstration mode.
DL3	The reference floor mark should be established when the robot is in its home position (perch) at a time when the laser indicating the beam central axes has been verified to be coincident with the radiation field center.
DL4	Prior to measuring the accelerator output, an accelerator and monitor unit chamber warm up irradiation of 6000 monitor units (MUs) for CyberKnife^®^ models with open monitor unit chambers, and 3000 MU for sealed monitor unit chambers should be delivered.
DL5	The automated quality assurance test is a measurement similar to the Winston–Lutz test which assesses the pointing accuracy for two orthogonal beam directions using a hidden high density target and two orthogonal films. Prior to delivering this test, the x‐ray system should be warmed up. The accuracy of automated robotic couch motion in response to positioning requests should also be assessed (at a minimum qualitatively) during this test.
DL6	This qualitative test is meant to verify individual leaf calibration variation and sticking through visual inspection of a series of abutted rectangular fields covering the entire range of motion as described in AAPM task group report 50.^4^ Test to be performed daily or at a minimum each day the MLC is to be used for patient treatment. Quantitative analysis of these films is complicated by the lack of a flattening filter and intentional MLC defocusing used to reduce interleaf leakage on this system. For this reason, two films should be acquired for this test: one film with abutting fields (in which junctions are expected to be hot); and one film with one MLC bank offset by 0.25 mm at all junctions except for 0.5 mm at isocenter (which should have a cold junction).

**Table 2:** Monthly quality control tests

Designator	Test	Performance	
		Tolerance	Action
Monthly			
ML1	Energy constancy (change in TPR or PDD ratio)	1%	2%
ML2	Accelerator output	2%	3%
ML3	Intracranial and extracranial isocentric end‐to‐end test; scheduled to cycle through each clinically used tracking method, path, and collimation system (fixed, Iris™, and InCise™ MLC)	Error in any direction: 0.75 mm (static); 1 mm (Synchrony^®^)	Radial error: 1 mm (static); 1.5 mm (Synchrony^®^)
ML4	Nonisocentric patient specific quality assurance; scheduled monthly to cycle through each clinically used tracking method, path, and collimation system at least quarterly (fixed, Iris™, and InCise™ MLC)	n/a	5% / 2 mm (static); 5% / 3 mm (Synchrony^®^)
ML5	Iris™ field size verification	±0.3 mm	±0.5 mm
ML6	Garden fence MLC test	n/a	±0.5 mm for 95% of leaf positions; <2 failures/leaf
ML7	Low contrast details visibility and spatial resolution of amorphous silicon detectors	n/a	Reproducible
ML8	Records	Complete	

**Notes on monthly tests**	
ML1	Energy constancy measurements shall be made by measuring the ratio of tissue phantom ratio (TPR) or percentage depth dose (PDD) at two different depths greater than d_max_ and separated by a minimum of 10 cm (e.g., TPR_20,10_) using the reference field size (typically a 60 mm diameter cone or a 10 × 10 cm^2^ field for systems equipped with the InCise^TM^ MLC).
ML2	Using a dosimetry system calibrated against the local secondary standard, the output of the linac shall be checked against annual reference dosimetry.
ML3	One cranial and one extracranial end‐to‐end test shall be performed monthly scheduled to cycle through each clinically used tracking method, path and collimation systems (fixed, Iris™, and InCise™ MLC). This test assesses the overall spatial targeting accuracy of the integrated CyberKnife^®^ system for multiple beams delivered isocentrically. This test uses the relative dose delivered to two orthogonal films in a phantom geometry capable of reproducing features necessary for each tracking algorithm (e.g., moving fiducials for Synchrony^®^ or simulated bony features for skull or spine tracking).
ML4	One cranial and one extracranial delivery quality assurance test for nonisocentric patient plans shall be performed monthly scheduled to cycle through each clinically used tracking method, path and collimation system. This test assesses the dosimetric accuracy of the entire system for nonisocentric delivery. An appropriate detector shall be used for the field sizes and dose gradients within the plan to be measured. For example, for plans using small collimators (≤10 mm in diameter), the use of radiochromic film is strongly recommended. Action levels for these tests refer to >90% pass rate for pixels in the high dose region (>50% isodose) for a gamma metric with the stated absolute dose percent difference/distance‐to‐agreement criteria.
ML5	This test verifies the field size long‐term stability and reproducibility of the Iris™ variable collimator by comparing to a baseline set of measurements immediately following beam data collection. Radiochromic film or equivalently high spatial resolution detector should be used. A smaller subset of field sizes may be tested each month provided that, at a minimum, all clinically used field sizes are rotating through quarterly.
ML6	For systems equipped with the InCise™ MLC, the “Garden fence” MLC test^5^ shall be performed monthly to provide quantitative information about MLC calibration for individual leaves.
ML7	Images of a phantom intended for planar kV image quality shall be acquired monthly and compared to a baseline. Ensure that an x ray warm‐up has been performed prior to image acquisition. The phantom's low and high contrast structures should be oriented perpendicular to the imaging systems’ central axes (i.e., in a stand rotated 45 degrees with respect to the horizontal direction). At a minimum, low‐contrast visibility and high‐contrast spatial resolution features should be assessed qualitatively (e.g., maximum number of low‐contrast objects visible, maximum number of line pairs/mm visible) and compared to the baseline.
ML8	Documentation relating to the daily quality control checks, preventive maintenance, service calls, and subsequent return to service must be complete, legible, and the operator identified.

**Table 3:** Quarterly quality control tests

Designator	Test	Performance	
		Tolerance	Action
Quarterly			
QL1	Beam symmetry	2%	3%
QL2	Beam profile shape compared to beam data	2% / 2 mm	3% / 2 mm
QL3	Imager alignment center	0.5 mm	1 mm

**Notes on quarterly tests**	
QL1–2	The beam shape and beam symmetry should be compared to values obtained during commissioning, typically using the 60 mm diameter collimator and a high resolution detector such as radiochromic film. If film is used, agreement with commissioning data refers to a >90% pass rate for a gamma metric with the stated absolute dose percent difference/distance‐to‐agreement criteria. Alternatively, if a detector array is used, it is recommended that at least three radial locations across 80% of the nominal field width are evaluated for this check with action and tolerance levels based on the stated percent differences from a baseline acquired using the same device immediately following beam data collection.
QL3	The alignment of the imaging system with respect to the isocrystal shall be assessed by acquiring images of the isopost and measuring the distance between the centroid of the crystal and center of the imager field of view for each imaging panel.

**Table 4:** Annual quality control tests

Designator	Test	Performance	
		Tolerance	Action
Annual			
AL1	Reference dosimetry	1%	2%
AL2	TPR or PDD and output factors for each clinically used collimation system	1%	2%
AL3	Radial profile constancy	1%/1 mm	2%/2 mm
AL4	Dose output linearity to lowest MU/beam used	1%/1 MU (0.5 MU end monitor effect)	2%/2 MU (1 MU end monitor effect)
AL5	Verify relative location of the central axis beam laser to the radiation central axis to ensure it has not changed from the baseline and is coincident	Change from baseline: 0.5 mm	Coincidence of laser and central axes: 1 mm
AL6	Verification of the second order path calibration	n/a	Each node < 0.5 mm RMS < 0.3 mm
AL7	Run Synchrony^®^ end‐to‐end test with at least 20° phase shift; analyze penumbra spread compared to static delivery	Radial Error: 1.0 mm 2 mm change in penumbra	Radial Error: 1.5 mm 3 mm change in penumbra
AL8	InCise™ MLC Leaf transmission	0.5%	1%
AL9	InCise™ MLC Leaf leakage between leaves	0.5%	1%
AL10	InCise™ MLC Transmission between abutting leaves	0.5%	1%
AL11	InCise™ MLC leaf alignment with jaws	0.5^o^	1.0^o^
AL12	Imager kVp, mA and timer accuracy, exposure linearity, exposure reproducibility	n/a	Reproducible
AL13	Quantitative assessment of contrast, noise, and spatial resolution of amorphous silicon detector	n/a	Reproducible
AL14	Independent review and update of quality assurance references	Complete	

**Notes on annual tests**	
AL1	A full absolute dosimetry output calibration based on an internationally accepted protocol such as AAPM TG‐51^6^ must be performed annually. Systems not equipped with the InCise™ MLC are not capable of producing a 10 × 10 cm^2^ field and therefore beam quality metrics necessary to determine *k*_Q_ for the fictitious reference field must be estimated using 60 mm cone data with equivalent field size corrections and standard reference data such as BJR supplement 25. A secondary independent check using optically stimulated luminescent dosimeters (OSLD) or thermoluminescent dosimeters (TLD) program through an accredited dosimetry calibration lab (ADCL) is also recommended.
AL2	Beam data checks of TPR (or PDD) and output factors for at least three field sizes for each clinically used collimator system in clinical use including the largest and smallest field size used. Care should be taken to use the same detector as that used during commissioning; particularly PDD data and output factors are especially sensitive to detector design for small fields.
AL3	Radial profile measurements should be made for at least three field sizes for each clinically used collimator system in clinical use including the largest and smallest field size used.
AL4	Dose output linearity and end monitor effect are verified annually including the lowest MU beam permitted clinically (typically 5 MU although the planning system allows 1 or 2 MU).
AL5	Coincidence of the central axis beam laser and radiation central axis should be better than 1 mm (action level) and should not have changed from the baseline by more than 0.5 mm (tolerance level). Measurements at two different distances from the radiation source (e.g., in the birdcage assembly and on the floor) are recommended assessing orthogonality of the laser. This test should also be performed before any verification or recalibration of the first‐ and second‐order path calibrations.
AL6	Verification of the second‐order path calibration for all clinically used pathsets shall be performed annually. If this test is performed as part of the preventative maintenance or during a path calibration, the verification reports will be reviewed by a medical physicist as part of annual quality control. In the current version of the software (CyberKnife^®^ System V10.X or less), this is only possible in the service mode with the help of an experienced service engineer.
AL7	A Synchrony^®^ end‐to‐end test run with at least a 20° phase shift between the LED markers and internal motion provides a measure of the system's ability to correct for a lag between internal and external motion. The 20° phase shift can easily be achieved via setting on the vendor supplied Synchrony^®^ quality assurance tool motion phantom.
AL8–11	The leakage, transmission characteristics of the InCise™ MLC shall be compared to baseline values determined at the time of commissioning as well as assessment of the alignment of MLC leaves.
AL12	Test methods for kVp, mA, and timer accuracy, exposure linearity, and reproducibility shall be performed annually and following any significant change to the kV imaging system done during preventative maintenance or machine service. These data should be acquired by firing the x‐ray tubes one at a time (currently not possible through the CyberKnife^®^ console standard interface) using a range of imaging parameters for both focal spot sizes. Ensure that an x‐ray warm‐up has been performed prior to image acquisition. Procedure development may require the assistance of a field service engineer.
AL13	A more quantitative version of the monthly image quality test shall be performed annually and following any significant service to the kV imaging system. Ensure that an x‐ray warm‐up has been performed prior to image acquisition. Raw or processed images from each panel may be extracted from the system using the treatment fraction download (TFDL) utility on the treatment console computer using a terminal emulator and ssh‐based file transfer program (e.g., PuTTY).
AL14	To ensure redundancy and adequate monitoring, a second qualified medical physicist must independently verify the implementation, analysis, and interpretation of the quality control tests at least annually. Quality assurance references should be updated annually as needed including acquiring new CT scans and plans for phantoms used in end‐to‐end testing.

## 3 RELATED TECHNICAL QUALITY CONTROL GUIDELINES

In order to comprehensively assess CyberKnife^®^ Technology performance, additional guideline tests, as outlined in related CPQR Technical Quality Control (TQC) guidelines must also be completed and documented, as applicable. Related TQC guidelines, available at cpqr.ca, include:

- Safety Systems
- Major Dosimetry Equipment

## CONFLICT OF INTEREST

The Ottawa Hospital Cancer Centre holds research agreements with Accuray Incorporated. Eric Vandervoort is the principal investigator for a research grant funded by Accuray Incorporated.
